# Complete remission of nephrotic syndrome in a young woman with anti-LRP2 nephropathy after immunosuppressive therapy

**DOI:** 10.1186/s12882-020-02027-w

**Published:** 2020-08-24

**Authors:** Xiaoye Zhu, Lingxue Tu, Shaojun Liu, Huaizhou You, Jun Xue, Chuanming Hao

**Affiliations:** 1 Division of Nephrology, Huashan Hospital, Fudan University, 12 middle Wulumuqi Road, Shanghai, 200040 China; 2 Department of Renal Medicine, the Fourth Affiliated Hospital of Xinjiang Medical University, Xinjiang, China

**Keywords:** Anti-LRP2 nephropathy, Anti-brush border antibody, Kidney biopsy, LDL receptor-related protein 2, Renal pathology, Megalin

## Abstract

**Background:**

Anti-low density lipoprotein receptor-related protein 2 (LRP2) nephropathy/anti-brush border antibody (ABBA) disease is a disorder characterized by acute tubulointerstitial injury associated with circulating antibodies to kidney proximal tubular brush border protein LRP2/megalin. Patients are typically elderly and present with acute kidney injury and subnephrotic proteinuria. They progress to end-stage renal disease with poor response to immunosuppressive therapies.

**Case presentation:**

We report a case of a 29-year-old Chinese woman, who presented with nephrotic syndrome with normal kidney function. Kidney biopsy showed no obvious tubular injury or interstitial inflammation. Positive immunoglobulin G (IgG) staining was revealed along the brush border of proximal tubular cells. Anti-LRP2 antibody was identified in serum, consistent with a diagnosis of anti-LRP2 nephropathy. The patient achieved complete remission after receiving prednisone and cyclophosphamide.

**Conclusions:**

Anti-LRP2 nephropathy can also present as nephrotic syndrome in young patients and complete remission from nephrotic syndrome may be achieved after immunosuppressive therapy.

## Background

Anti-brush border antibody (ABBA) disease is a disorder characterized by acute tubulointerstitial injury associated with circulating antibodies reactive to kidney proximal tubular brush border. ABBA was first described in 1981 and a total of 30 cases have been reported in the literature [1–8]. Circulating antibodies reactive to normal human kidney proximal tubular low-density lipoprotein (LDL) receptor-related protein 2 (LRP2), also known as megalin, have been identified as being responsible for the disorder [4]. The largest cohort study, by Larsen et al, described 27 ABBA patients (mean age 70, range [49–90] years) who presented with acute kidney injury (AKI) (mean creatine 3.6 mg/dL, range [1.3–8.8] mg/dL) and subnephrotic range proteinuria [8]. Kidney biopsies demonstrated acute tubular injury with variable but mild tubulointerstitial inflammation. Tubular basement membrane (TBM) stained positive for immunoglobulin G (IgG) in all cases and LRP2 antigen was found to be co-localized with IgG along the TBM. Some of the patients had positive staining along the apical border of the proximal tubules. Most of the patients also had segmental subepithelial immune deposits in the glomeruli [8]. As the optimal treatment for anti-LRP2 nephropathy was unknown, prednisone, cyclophosphamide (CTX) or rituximab were used. At a mean time of 9.2 months follow-up, one patient had achieved complete recovery, 11 patients had persistent disease, 3 patients progressed to ESRD and 6 were deceased [8].

Here we present a case of anti-LRP2 nephropathy in a young Chinese woman who presented with nephrotic syndrome and normal kidney function, and achieved complete remission after prednisone and CTX treatment.

## Case presentation

A 29-year-old woman from Xinjiang, China, presented with pitting edema of the lower extremities, without face or eyelid swelling, for a week and was admitted for further evaluation. The patient had no medical history of diabetes, hypertension, autoimmune diseases, infections, drugs, etc. No family history of renal diseases was reported. Her vital signs were normal and her physical examination was unremarkable except for the edema in lower extremities.

At the time of presentation, laboratory tests showed: urinary protein 4+, red blood cells 14.4/high-power field (HPF), white blood cells 13.8/HPF; 24 h urine protein, 3.8 g; serum albumin, 13.5 g/l; serum creatinine, 0.5 mg/dL; hemoglobin, 8.4 g/l; mean corpuscular volume (MCV), 67.3 fl (reference range, 82–100); mean corpuscular hemoglobin (MCH), 17.2 pg (reference range, 27–34); mean corpuscular hemoglobin concentration (MCHC), 255 g/L (reference range, 316–354); hematocrit, 0.329 L/L (reference range, 0.35–0.45); reticulocyte count, 0.83% (reference range, 0.5–1.5), serum iron, 6.6 umol/L (reference range, 7–30), while white blood cell counts and platelets were normal. Blood lipids were: cholesterol 8.49 mmol/L, triglyceride 1.77 mmol/L, low-density lipoprotein (LDL) 5.72 mmol/L, high-density lipoprotein (HDL) 1.38 mmol/L. Serologies, including anti-nuclear antibody, anti-double-stranded DNA, ANCA and anti-glomerular basement membrane (GBM), were negative. C3 and C4 levels were normal. Her hepatitis B and C virus and human immunodeficiency virus (HIV) were negative.

**Fig. 1:**
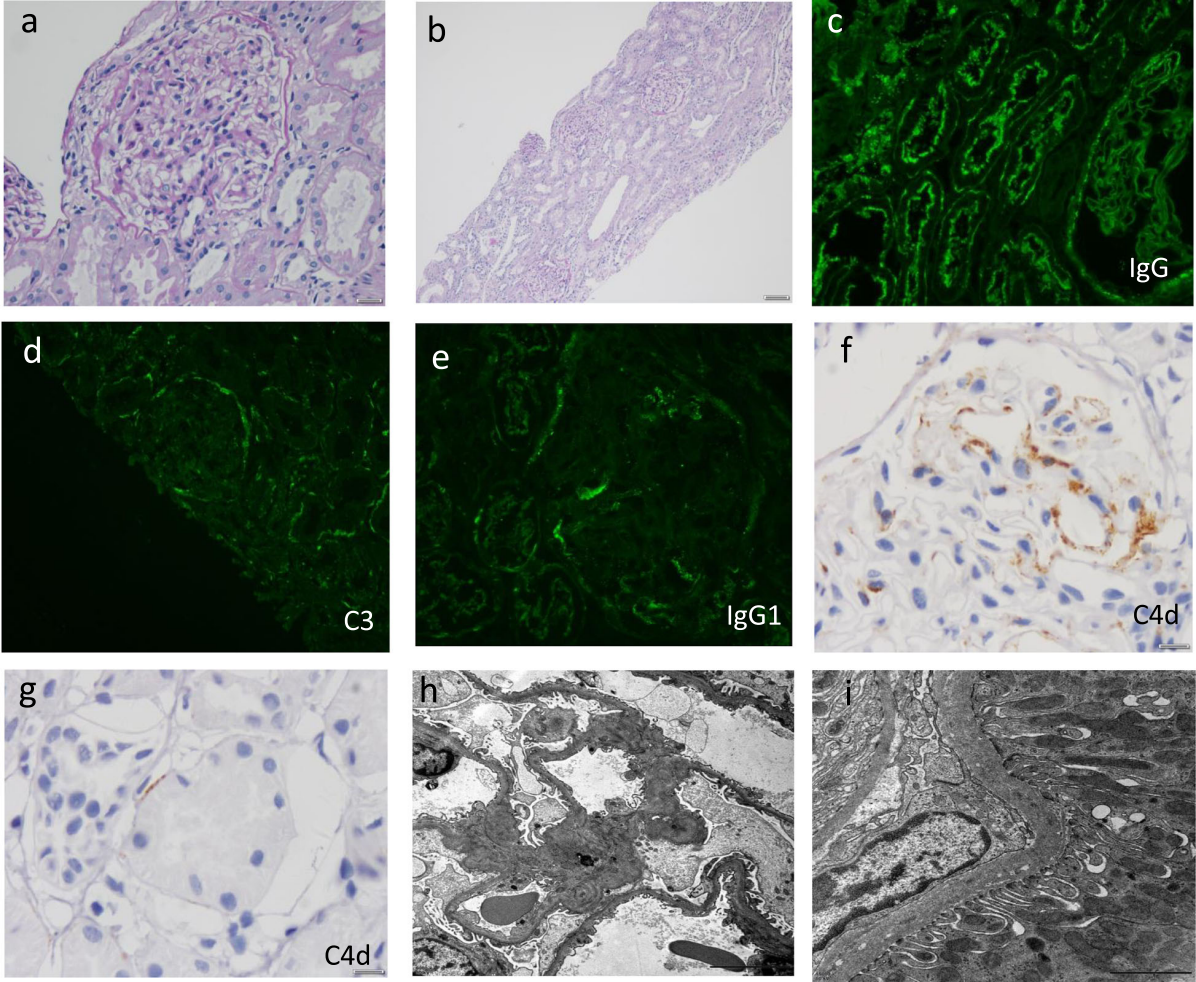
Characteristic morphology of kidney biopsy in this case. **a**-**b** Glomeruli were without morphologic abnormalities and no obvious tubular injury. (**a**: Periodic acid Schiff PAS, original magnification × 400; **b**: Periodic acid Schiff PAS, original magnification× 100). **c** IgG staining along the brush border of proximal tubular cells, some TBMs, Bowman’s capsule and small segmental granular deposits along GBM (direct immunofluorescence, original magnification × 400). **d** C3 staining along TBMs, Bowman’s capsule and small segmental granular deposits along GBM (original magnification × 400). **e** IgG1 was present in the brush border of proximal tubular cells, TBM and GBM (original magnification × 400). **f**-**g** Immunohistochemistry for C4d revealed segmentally sparse deposits along GBM (**f**) and TBM (**g**) (original magnification × 600). **h**-**i** Electron microscopy (**h**) of glomeruli presented foot process effacement without electron-dense deposits (original magnification × 6000). **i** Small granular electron-dense deposits within the proximal TBMs (original magnification × 15,000)

Light microscopy (LM) examined 14 glomeruli, none of which were globally sclerosed. Glomeruli were without morphologic abnormalities by LM (Fig. 1a). No tubular injury, as manifested by loss of brush border, blebs, vacuoles and regenerative changes, were observed. No tubulitis, interstitial inflammatory infiltration or fibrosis (Fig. 1b), and no arteriosclerosis or arteriolar hyalinosis were found. There was no vasculitis or thrombi.

**Fig. 2:**
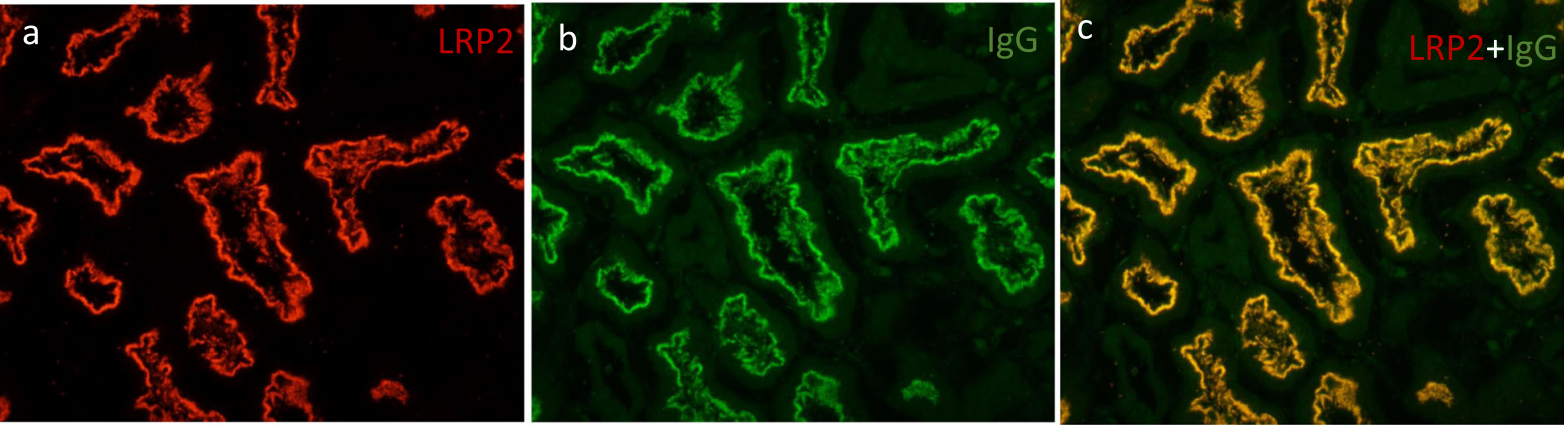
Colocalization of LRP2 and IgG on the patient’s renal biopsy sample. **a**-**c** Immunofluorescence experiments showed positive staining along the apical membrane of the proximal tubules for: **a** LRP2 using a rabbit polyclonal antibody and **b** IgG. **c** Strong colocalization of LRP2 and IgG along the apical membrane of the proximal tubules

**Fig. 3:**
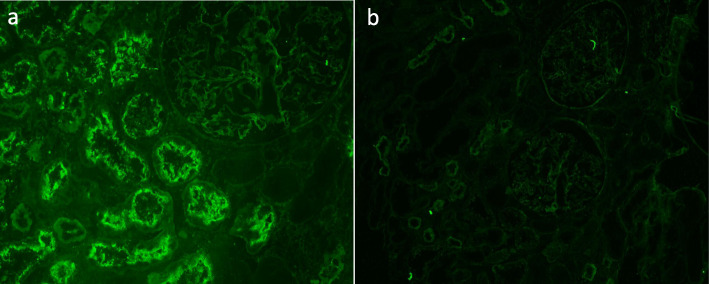
Immunofluorescence experiments using anti-LRP2 nephropathy and control patient serum. **a** Indirect immunofluorescence using the anti-LRP2 nephropathy patient’s serum on a normal human kidney section, showing positive IgG staining along the proximal tubule brush border. **b** Indirect immunofluorescence using the IgA nephropathy patient’s serum on a normal human kidney section, showing negative IgG staining

**Fig. 4:**
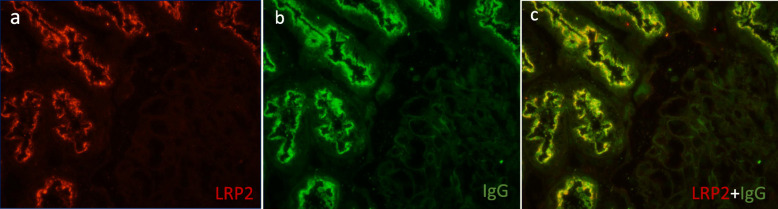
Immunofluorescence performed on cryosections of normal human kidney tissue. **a** Staining for LRP2 using a rabbit antibody along the apical membrane of the proximal tubular epithelium. **b** Indirect immunofluorescence of serum from a patient with anti-LRP2 nephropathy on a normal human kidney. **c** Strong colocalization of LRP2 and serum antibodies

Immunofluorescence (IF) analysis revealed deposits of IgG along the brush border of proximal tubular cells and some segments of TBM and Bowman’s capsule (Fig. 1c), with equal staining of κ and λ chains. A few segmental granular deposits of IgG were also observed along the GBM. Phospholipase-A2 receptor (PLA2R) staining was negative. Staining for IgG subclasses showed that IgG1 was present in the brush border of proximal tubular cells, TBM and GBM, with a distribution pattern consistent with IgG staining (Fig. 1e). IgG2, IgG3 and IgG4 were not detected. C3 stained positive along TBMs and Bowman’s capsule, with no obvious reactivity along tubular brush borders (Fig. 1d). IgA, IgM and C1q staining was negative. Immunohistochemistry for C4d revealed segmentally sparse deposits along GBM and TBM (Fig. 1f-g). LRP2 immunofluorescence (using rabbit antibody from Thermofisher, PA5–64182) was positive along the proximal tubular brush border and colocalized with IgG. No LRP2 was found along TBM and GBM (Fig. 2). Indirect immunofluorescence using the patient’s serum (at a titer of 1:100) on a normal human kidney section showed positive staining along the proximal tubule brush border, while sera from patients with minimal change disease (MCD) and IgA nephropathy or from healthy subjects showed negative staining (Fig. 3). Co-staining showed that the fluorescence from the patient’s serum colocalized with the LRP2 on a normal human kidney section (Fig. 4). Detailed methods are provided in the supplementary material.

Electron microscopy showed small granular electron-dense deposits within the proximal tubule TBMs (Fig. 1i), and podocyte foot process effacement. No deposits were found in subepithelial areas nor in the Bowman’s capsule (Fig. 1h).

The patient was treated with predinisolone plus intravenous CTX. The predinisolone was given at 1 mg/kg for 2 months, then was gradually tapered over 7 months. Intravenous CTX was used at a dose of 1 g, monthly for 6 months, then 1 g again 3 months later. Total CTX load was 7 g. Three months after the treatment, the patient achieved a complete remission, with negative proteinuria and a serum albumin level of 35 g/l. The serum creatinine was 0.5 mg/dl. The ABBA titer in serum decreased from 1:100 to 1:10 9 months after the therapy. Her hemoglobin increased to 12.2 g/l after oral iron therapy.

## Discussion and conclusions

Here we describe a young woman with anti-LRP2 nephropathy, who presented with (1) nephrotic syndrome with normal renal function; (2) IgG (predominantly IgG1) staining along the brush border of proximal tubular cells, TBM and occasionally along GBM; (3) anti-LRP2 antibodies in the serum; and (4) complete remission of nephrotic syndrome 3 months after steroid and cyclophosphamide treatment.

There are few reports of anti-LRP2 nephropathy with confirmed serum ABBA (anti-LRP2 antibody) [1–8]. In 2016, a case report described a patient with tubulointersitial nephritis with immune complexes in the TBM and occasional epimembranous deposits. The authors found a strong binding of IgG to the brush border of proximal tubules, using the patient’s serum in indirect IF, on cryosections of normal kidneys, and confirmed the diagnosis of ABBA disease [3]. In 2018, a series of 10 cases of ABBA was reported. All had granular IgG and C3 staining along TBMs and Bowman’s capsule, and half of them had IgG and C3 along the apical border of the proximal tubules. The target antigen of the circulating ABBA autoantibodies was identified as LRP2, and it was proposed that the condition could be more specifically termed anti-LRP2 nephropathy. In their study, a polyclonal rabbit antibody did not recognize LRP2 within TBM deposits, but a mouse mAb showed positive staining in all patients [4]. Immunofluorescence for LRP2, using the rabbit antibody, in our patient’s biopsy also failed to show TBM deposits, probably because the specific antigenic epitopes were not exposed.

Although anti-LRP2 nephropathy is a rare disorder, and it is easily diagnosed if IgG stains positive along the brush border of proximal tubular cells, but it will be overlooked when only positive IgG staining of TBMs is found. Detection of antibodies reactive to normal human kidney proximal tubular brush border by indirect IF with a patient’s serum will further support the diagnosis of anti-LRP2 nephropathy. The pathologist should pay attention to the possibility of this discorder and perform prompt confirmatory serum studies after detection of any IgG-containing TBM immune deposits. In addition to TBM deposits, Larsen et al also reported that most of the patients had segmental subepithelial immune deposits in their glomeruli [4]. Anti-LRP2 nephropathy should be considered if segmental subepithelial immune deposits in glomeruli combined with TBM deposits are found.

In our case, the IgG1 subclass was predominant, while IgG4 was negative. In contrast to published cases, our case showed no obvious tubular injury and interstitial inflammation by LM with normal kidney function. Interestingly, Rosales et al. reported a case with ABBA disease, whose IgG1 was co-dominant with IgG4 along proximal TBM. This patient received a renal transplant and the disease recurred in the allograph 7 weeks after transplantation, with IgG1 being the predominant deposit [3]. There is also evidence that suggests IgG subclass switching in membranous nephropathy (MN), with IgG1 deposits only in the early stage of MN, followed by IgG4 deposition [9]. Although the precise reasons for the discrepancies in IgG subclass deposition between our patient and previous reported cases is not clear, we speculate that limited tubular injury and interstitial inflammation, with IgG1 predominant along the proximal tubular brush border and less TBM staining, suggests early stage anti-LRP2 nephropathy.

In the present report, our patient had profound nephrotic syndrome with podocyte foot process effacement. In fact, in published cases of anti-LRP2 nephropathy, although all patients presented as AKI, one patient also had nephrotic proteinuria that was as high as 14 g per day [8]. The mechanism of nephrotic proteinuria in anti-LRP2 nephropathy is not known. Drachenberg et al reported that C4d was negative in MCD, but positive in immune-complex glomerulonephritis [10]. Therefore, in our patient, the presence of C4d and IgG along the capillary loop does not seem to support MCD. However, it was difficult to rule out the possibility that anti-LRP2 nephropathy coexisted with MCD. The antigen(s) in these immune complexes along the capillary loop are unclear. Interestingly, trace amounts of LRP2 have been reported in the parietal and visceral epithelium under normal conditions [11]. Whether this antibody was also directed against LRP2 remains to be clarified.

IgG4-related disease represents a recently recognized group of multi-organ diseases characterized by a high level of serum IgG4 and dense infiltration of IgG4-positive cells into multiple organs. Tubulointerstitial nephritis was the major finding in renal parenchymal lesions [12]. Although the major pathological feature of this disease is tubulointerstitial nephritis, membranous nephropathy is the most frequent feature among glomerular injuries. There is a morphologic overlap with anti-LRP2 nephropathy, since both exhibit interstitial inflammation and glomerular deposits in addition to the tubular deposits. However, renal biopsy of our patient does not seem to support IgG4-related kidney injury - less intense interstitial inflammation, lack of the characteristic storiform fibrosis of IgG4-related disease and strong tubular brush border staining for IgG by IF.

Since anti-LRP2 nephropathy is an autoimmune disease, prednisone, cyclophosphamide, and rituximab have been used in reported cases, but 50% of patients required a kidney transplant or died at a median follow-up time of 7 months [4]. However, our patient with nephrotic syndrome recovered quickly after treatment with prednisone and cyclophosphamide and her renal function remained normal throughout the disease course up to 9 months of follow-up.

In summary, we report a young female with nephrotic syndrome and normal renal function. Although our patient lacked acute tubular injury and interstitial inflammation by LM, IgG positive staining along the brush border of proximal tubular cells suggested the presence of anti-LRP2 nephropathy. Detection of anti-LRP2 antibody in serum eventually confirmed the diagnosis. The patient achieved a complete remission from nephrotic syndrome after immunsuppressive therapy.

## Supplementary information

**Additional file 1.**

## Data Availability

The datasets related to this case report are available from the corresponding author.

## Abbreviations

ABBA: Anti-brush border antibody
LRP2: Low-density lipoprotein receptor-related protein 2
TBM: Tubular basement membrane
IgG: Immunoglobulin G
CTX: Cyclophosphamide
GBM: Glomerular basement membrane
LM: Light microscopy
IF: Immunofluorescence
PLA2R: Phospholipase-A2 receptor
MCD: Minimal change disease
AKI: Acute kidney injury
