# Supplementary material for: Complete remission of nephrotic syndrome in a young woman with anti-LRP2 nephropathy after immunosuppressive therapy
Source: BMC Nephrol. 2020 Aug 24;21:364. doi: 10.1186/s12882-020-02027-w (PMC7446201; doi:10.1186/s12882-020-02027-w)
Supplement: Supplementary file 1 — Additional file 1. [file 12882_2020_2027_MOESM1_ESM.pdf]

Supplementary method

Figure2 : Co-immunostaining with LRP2/Megalin and IgG on renal biopsy tissue:

1) Cut paraffin tissue section at 2um.

2) Oven dry at 37°C overnight.

3) Deparaffinize: Xylene for 10 min (×2), ethanol 100% for 5 min (×2), 95% for 5 min.

4) Antigen retrieval was performed by microwave with EDTA repair solution for 20 minutes, and cooled to room temperature.

5) Wash slides with PBS for 5 min (×3).

6) Incubate with rabbit anti-human LRP2 antibody (Thermofisher PA5-64182) at a dilution if 1:200 at 37℃ for 1h.

7) Wash slides with PBS for 5 min (×3).

8) Incubate with Cy3 conjugated anti-rabbit IgG (secondary antibody, JACKSON, 715-165-152) at a dilution of 1:50 at room temperature for 30min.

9) Wash slides with PBS for 5 min (×3).

10) Add FITC-labeled antibody (polyclonal rabbit anti-human IgG-FITC, DAKO, F020202-2) at a dilution of 1:50 and incubate at room temperature for 1 h.

11) Wash slides with PBS for 5 min (×3).

12) Cover the tissue section with coverslip using glycerin and examine under fluorescence microscopy.

Figure3 : Indirect immunofluorescence of serum from a patient with anti-LRP2 nephropathy:

1) Cut the cryosection of normal human kidney tissue at 2um.

2) Wash slides with PBS for 5 min.

3) Directly add the serum from the patients to the kidney section, at a concentration of 1 ：10, 1 ：20, 1 ：50, 1 ：100, and 1:200 diluted with PBS and incubate at 37℃ for 1h.

4) Wash slides with PBS for 5 min (×3).

5) Incubate with secondary antibody (polyclonal rabbit anti-human IgG/FITC, DAKO, F020202-2) at a dilution of 1:50 at room temperature for 30min.

6) Wash slides with PBS for 5 min (×3).

7) Cover the tissue section with coverslip using glycerin and examine under fluorescence microscopy.

The maximum dilution that showed a positive result is the titer of the patient's serum.

Figure 4 ：Indirect immunofluorescence of serum from a patient with anti-LRP2 nephropathy on the normal human kidney, and co-labeling with of LRP2/megalin:

1) Cut the cryosection of normal human kidney tissue at 2um.

2) Wash slides with PBS for 5 min.

3) Directly add the serum from the patients to the kidney section, at a concentration of 1 ：10，1 ：20， 1 ：50， 1 ：100， and 1:200 diluted with PBS and incubate at 37℃ for 1h.

4) Wash slides with PBS for 5 min (×3).

5) Incubate with secondary antibody (polyclonal rabbit anti-human IgG/FITC, DAKO, F020202-2) at a dilution of 1:50 at room temperature for 30min.

6) Wash slides with PBS for 5 min (×3).

7) Add rabbit anti-human LRP2 antibody (Thermofisher PA5-64182) at a dilution of 1:200 and incubate at 37°C for 1 h.

8) Wash slides with PBS for 5 min (×3).

9) Incubate with Cy3 conjugated anti-rabbit IgG (secondary antibody, JACKSON, 715-165-152) at a dilution of 1:200 at room temperature for 1h.

10) Wash slides with PBS for 5 min (×3).

11) Cover the tissue section with coverslip using glycerin and examine under fluorescence microscopy.
